# Stuck-moving needle acupuncture myofascial trigger point to treat idiopathic frozen shoulder: study protocol for a randomized controlled trial

**DOI:** 10.1186/s13063-020-04799-w

**Published:** 2020-10-30

**Authors:** Yang Bai, Ying Wang, Bo Chen, Yinan Qin, Qianqian Lei, Hailong Zhao, Jingbao Lu, Qian Fan, Yali Wang, Hongbo Song, Maomao Cheng, Wei Wang, Shengen Hu, Yuan Hao Du, Tian Xia

**Affiliations:** 1 The First Affiliated Hospital of Tianjin University of Traditional Chinese Medicine, Tianjin, 300193 China; 2 Tianjin University of Traditional Chinese Medicine, Tianjin, 300073 China; 3 Department of Acupuncture and Massage, Qingyang Hospital of Traditional Chinese Medicine, Qingyang, 745000 Gansu China; 4 Department of Reproductive Medicine, The First People’s Hospital of Nanyang, Nanyang, 473005 Henan China

**Keywords:** Stuck-moving needle acupuncture, The myofascial trigger point, Idiopathic frozen shoulders, Study protocol

## Abstract

**Background:**

There are evidence for the efficacy of acupuncture treatment for chronic shoulder pain, however, it remains unclear the best acupuncture modes for effective treatment. We compared the effect of the myofascial trigger point (MTrp) stuck-moving needle acupuncture with that of common acupuncture treatments. Further, we evaluated the efficacy and safety of stuck-moving needle acupuncture for the MTrp in improving pain and range of motions in patients with idiopathic frozen shoulder. The aim of present study is to select an effective therapy for patients with idiopathic frozen shoulder.

**Methods:**

Randomized controlled trial will be conducted in the three clinical centers of Qingyang Traditional Chinese Medicine Hospital, Qingyang Xifeng district People’s Hospital, and Qingyang Second People’s Hospital in China from February 2020 to January 2021. One hundred and eight frozen shoulder patients will be recruited and randomized into one of three groups in a 1:1:1 ratio of the stuck-moving needle acupuncture group, common acupuncture control group, and physical exercise control group. This trial will include a 1-week baseline period, a 3-week treatment period, and a 12-week follow-up period. During the 3 weeks of the treatment period, patients will receive nine sessions of acupuncture. The primary outcome will be related to change in the Visual Analogue Scale (VAS) and measurement of range of joint motion (ROM) from the baseline period to the 12-week follow-up period. Secondary outcome measures will include measurement of pressure pain threshold (PPT), pressure pain tolerance (PTT), Oxford Shoulder Score (OSS), 36-item short form survey, and patient satisfaction evaluation. Adverse events also will be recorded for safety assessment.

**Discussion:**

The results of this trial will allow us to compare the difference in efficacy between stuck-moving needle acupuncture MTrP with that of common acupuncture treatments. The findings from this trial will be published in the peer-reviewed journals.

**Trial registration:**

Acupuncture-Moxibustion Clinical Trial Registry (ChiMCTR1900002862) and Chinese Clinical Trial Registry (ChiCTR1900028452). Registered on 22 December 2019. http://www.chictr.org.cn/showproj.aspx?proj=47354

## Background

### Epidemiology and current management

The shoulder joint is the joint which involves the largest range of human activities. It can perform complicated work and exercise. Therefore, their significance is inevitable in daily work activities of the human being. Shoulder pain is a common disease with high morbidity in patients attending daily outpatient clinics [1–4]. The frozen shoulder is a type of shoulder disease in which the active and passive movements of the shoulder joints are significantly restricted for no apparent reasons. The study carried out by Codman [5] in 1934 pioneered the term “Frozen Shoulder.” Later, the American Academy of Shoulder and Elbow Surgeons defined the disease as “adhesive arthritis of the glenoid-humeral joint” [6]. At present, the two names such as “frozen shoulder” and “adhesive arthritis” are used worldwide. Generally, middle-aged and elderly people in their 40s and 60s are most susceptible to the frozen shoulder disease. In the normal population, occurrence of this disease is about 2 to 5%, whereas diabetic patients are more susceptible to the occurrence rate as high as 20% [7]. The frozen shoulder is mainly divided into idiopathic frozen shoulder and secondary frozen shoulder. Idiopathic frozen shoulder is considered as a diagnosis for all cases in which specific trauma, known comorbidity, or underlying etiology cannot be identified. The clinical characteristics can be defined with shoulder pain and mechanical restriction in all planes of movement of the glenohumeral joint [8]. It is considered to be a self-limiting disease that can resolve within 1 to 3 years. However, shoulder pain and limited mobility in some people may take years and some carry it for life-long [9]. Various studies and researches have shown that 20 to 50% of patients may continue to develop persistent symptoms [10]. The previous studies were carried out with continuous follow-up of 41 frozen shoulder patients for the period of 5–10 years; however, recovery was observed in only 39% of the patients. The long-term pain and limited mobility of most patients seriously affected their normal daily life [11]. It requires a management plan differing from other shoulder conditions [12]. Efforts to find out effective therapies to relieve pain and increase the range of motion are essential to improve the quality life of patients suffering from frozen shoulder.

### Rationale for the use of intervention

Idiopathic frozen shoulder treatment methods are generally divided into three types: operative treatment, conservative management, and minimally invasive treatment [13]. The operative treatment includes arthroscopic capsular release and manipulation under anesthesia, while conservative management includes exercise therapy, superficial heat or cold, electrotherapy, and medication. Minimally invasive treatment includes acupuncture and injections [9, 13]. Recent numerous studies have focused on acupuncture treatments for idiopathic frozen shoulder, as it is safer than medical treatments and has fewer side effects, specifically to remedy for chronic shoulder pains [14, 15]. Based on previous studies, it has been suggested that the strong stimulation of acupuncture may enhance the effects of pain relief by triggering “diffuse noxious inhibitory controls” and may increase the secretion of endorphins by stimulating internal activity of the central nervous system [16, 17]. In addition, acupuncture can inhibit the inflammatory response of the local shoulder joint [18]. However, the acupuncture site, manipulation, and intensity of the stimulation affect the efficacy of acupuncture therapy [19]. The common acupuncture stimulates the acupoints with filiform needles. It is easy to operate and the patient suffers less pain, whereas stuck-moving needle therapy is different from common acupuncture in the needle instrument, manipulation, and stimulation site [20]. Stuck-moving needle applicator applies active motion to the stuck needles fixed at the myofascial trigger points to achieve a benign, suitable, and effective needle volume, eventually to get acupuncture effect. Consequently, stuck-moving needle therapy has higher requirement on the operator and has stronger acupuncture stimulation compared with common acupuncture. The myofascial trigger points (MTrp) have often been used in the treatment of myofascial pain syndrome. Acupuncture is a type of myofascial trigger point therapy which provides immediate relief of pain related with myofascial trigger point [21–23]. Moreover, results from a few studies suggested that the response to trigger points is greater than the response to treating traditional acupoints [24, 25]. These results suggest that the stuck-moving needle stimulation of myofascial trigger points might be effective for patients with idiopathic frozen shoulder. Our pilot study showed that stuck-moving needle was used to acupuncture myofascial trigger point and was effective in improving the pain and movement limitation of the patients with idiopathic frozen shoulder. Physical exercise programs are widely used to resolve the shoulder joint pain which ultimately limits the frozen shoulder, and considered as the effective basic therapy [14] which is also supported by clinical evidences [13]. This information permits the use of physical exercise programs as the basic therapy for a control treatment. The present study was carried out to compare the effectiveness of stuck-moving needle acupuncture Trp and common acupuncture practices.

### Rationale for the trial design

Currently, few studies report on the comparative effectiveness of using stuck-moving needle acupuncture MTrp for idiopathic frozen shoulder. However, clinical studies have reported the benefits of acupuncture [26], but lack the comparison and the effect of different methods. Thus, in this study, we designed a prospective randomized controlled trial to determine the clinical effectiveness of stuck-moving needle acupuncture MTrp and common acupuncture in reducing idiopathic frozen shoulder. Further, we compared them to physical exercise programs. Although the double-blind, placebo-controlled trial is the golden standard to assess a therapeutic effect, however, this method is difficult to use for manipulation technique of sham needling. With a needle stimulation, the process of manipulation for stuck-moving needle acupuncture MTrp and common acupuncture is easily distinguished. Thus, in this study, we have not included blinding and placebo. However, this project uses a blind method for evaluation. Efficacy assessments were performed by third parties, and they were unaware of the grouping situation. In addition to the outcome measurement choice, this study will also evaluate additional patient satisfaction and short form-36 questionnaire (SF-36), and the safety evaluation.

## Method/design

### Study design

**Fig. 1:**
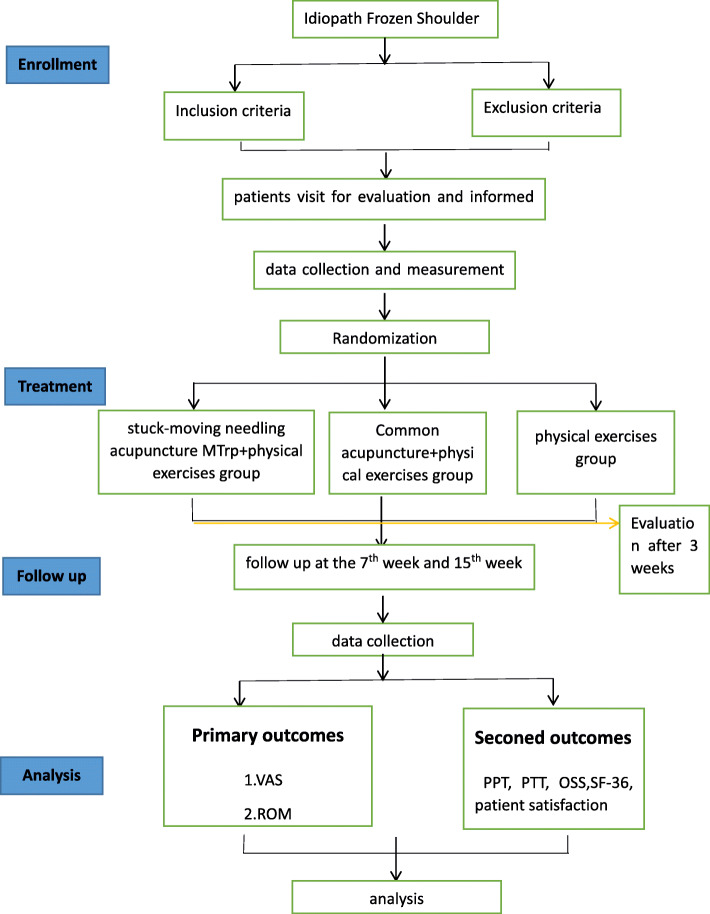
Trial procedure

The proposed study is a multicenter, prospective, randomized controlled trial that will compare the three currently available treatment options for patients with frozen shoulder. Study will include a 3-week treatment phase followed by a 12-week follow-up phase. Figure 1 shows the trial procedure, and Table 1 illustrates the trial schedule. A total of 108 FS patients will be recruited and randomized into one of three groups in a 1:1:1 ratio of the stuck-moving needle acupuncture group, common acupuncture control group, and physical exercise control group. Patients as a subject will be recruited from 3 clinical centers of Qingyang Traditional Chinese Medicine Hospital, Qingyang Xifeng district People’s Hospital, and Qingyang Second People’s Hospital from February 2020 to January 2021. All patients will be required to provide written consent to participate in the study. The study has been approved by the Ethics Committee of the Qingyang Hospital of Chinese Medicine (No.2019-12-001). Further, we have registered in the Acupuncture-Moxibustion Clinical Trial Registry (ChiMCTR1900002862) and Chinese Clinical Trial Registry (ChiCTR1900028452).

**Table 1:**
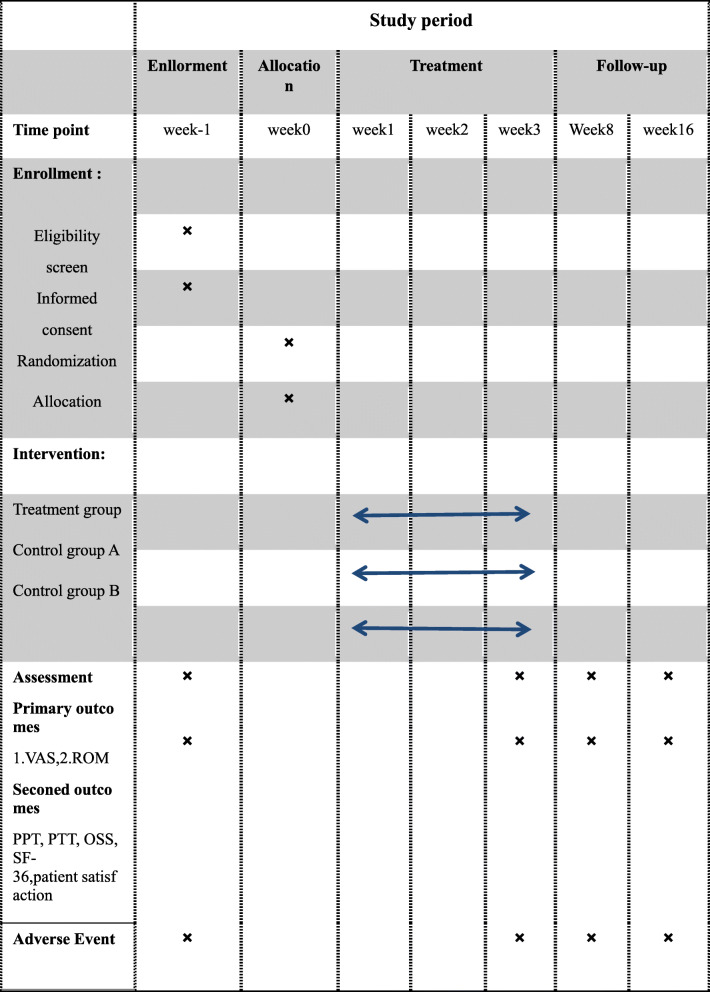
Schedule of enrollment, intervention and assessments for this study protocol

*VAS* Visual Analogue Scale, *ROM* measurement of range of joint motion, *PPT* pressure pain threshold, *PTT* pressure pain tolerance, *OSS* Oxford Shoulder Score, *SF-36* 36-item short form survey

### Participants

#### Participants criteria

The inclusion criteria for patient selection are as follows:

1. The age shoulder between 40 and 70 years old.
2. Shoulder pain is self-reported, whereas the pain range extends from the medial side of the shoulder to the biceps area or the outside of the deltoid muscle. The VAS score is between 7 and 8 points.
3. The onset of pain lasts for at least 3 months.
4. The stiff shoulder pain is a spontaneous attack, and the shoulder lifting or external rotation function is lost by 50%, and the OSS score is between 50 and 60 points.
5. Being able to actively cooperate with the doctor and sign the consent form to voluntarily receive acupuncture treatment.

#### Exclusion criteria

The exclusion criteria for patient selection are as follows:

1. The patient has received other treatments that may affect the effect indicators of this study, including oral painkillers for nearly 3 months and physical therapies such as acupuncture and massage.
2. The patient has severe life-threatening primary diseases related to liver and kidney, cardiovascular and cerebrovascular diseases, and mental illness.
3. Patients who are pregnant or breastfeeding which do not meet the inclusion criteria.
4. Exclude other factors related to the diseases such as the history of shoulder trauma within 1 month, shoulder tuberculosis and tumor, rheumatoid arthritis, rheumatic arthritis, gouty arthritis, shoulder subluxation of stroke sequelae, shoulder pain caused by cervical spondylosis, and reflex shoulder pain caused by heart or biliary tract disease.

Note: Patients who meet any of the above criteria will be excluded immediately.

## Recruitment, randomization, allocation concealment, and blinding

### Recruitment

Patients will be recruited via advertisements on hospital website and notice board of clinics.

### Random and allocation of hidden methods

The number of observed subjects will be allocated based on the actual situation of each center. The subjects in each center will be divided into three groups by random number table method and serial numbers will be allocated (such as 1, 2, 3, ..., *N*, this serial number is the ID of the subjects who will participate in the study). Then starting from any number on the random number table, the number of subjects will be selected in the sub-center. SAS (v9.4) software will be used to generate the random numbers corresponding to the above serial number. The number will be divided by 3; if the remainder is 0, then the subject will be included in the acupuncture myofascial trigger points’ group. If the remainder is 1, subject will be included in the common acupuncture group, and if the remainder is 2, then the subject will be included in the functional exercise group. According to the result of the grouping, the random card will be made, sealed in an opaque envelope, and will be kept by a responsible person. In the clinical implementation, the order in which the qualified subjects will be included in the test is in one-to-one correspondence with the sequence number on the envelope, and the envelopes will be opened and grouped according to the prompts on the random card.

### Blind method

This study will use the blind method for evaluation. Efficacy assessments will be performed by third parties who do not know the grouping situation. Further, all participants in the assessment will receive uniform training. Blind statistical analysis will be used in the data summary stage, that is, statistical analysts will be unaware of the grouping situation and content. Randomized staff, study researchers, operators, and statisticians will be kept unaware of the experiment blinded to ensure that subjects, evaluators, and statisticians do not understand the grouping situation.

## Intervention

Participants will be randomly allocated into one of three groups: A, the stuck-moving needling acupuncture MTrp group; B, the common acupuncture group; and C, the physical exercise group as the control group and the basic therapy for groups A and B. Three acupuncturists with at least 5 years of clinical experience in acupuncture practice will be recruited from Chinese Medicine Practitioners, and only these acupuncturists will be allowed to perform all acupuncture sessions. Several training sessions will be carried out prior to the commencement of the study which will ensure consistency in the skills of the acupuncturist. During the 3-week treatment period, patients will receive nine sessions of acupuncture (3 weekly cycles of one treatment every other day, followed by a 1-day break). One-time stuck-moving needles (0.40 mm × 40 mm/0.40 × 25 mm, the patent of Li Zhenquan produced, Beijing, China) and one-time acupuncture sterile needles (0.25 mm × 40 mm/0.25 × 25 mm, Hwato, Suzhou, China) will be used in the trial. All acupuncture treatments will be provided for free to improve adherence to the intervention protocol. Patient and acupuncturist signatures will be required after each acupuncture session to monitor adherence. Other treatments that may affect the effect indicators of this study will be prohibited, including oral painkillers for nearly 3 months and physical therapy such as acupuncture and massage.

### Group A: stuck-moving needle acupuncture MTrp treatment group

#### Selection and positioning method of shoulder joint myofascial trigger pain points

The selection of shoulder joint myofascial trigger pain points will be achieved by a dynamic search. Further, patients will be subjected to do three actions including the first one as abduction, lifting, and touching the head. The second one is forward flexion and internal rotation by touching the opposite shoulder. The third one is a posterior extension and touching patient’s back. These three actions can indicate the patient’s pain area. At the same time, the doctor will use his or her thumb to press these areas and confirms the muscle tension band. The compression can lead to localized pain and muscle spasm and strengthen the stimulation to induce the mentioned pain. Once the pain is induced, the doctor will ask the patient if the pain is similar to what he or she usually feels. If the pain is similar to the pain patient usually feels, then the doctor will place a pressure manometer on the main tenderness point to evaluate the pain point. Further, by placing the pressure manometer (Wagner Instruments, USA, specification model: FDK20) at the pain area, slowly pressure will be applied. Using this equipment, doctor can measure the tenderness threshold and can remove the pain points below the tenderness threshold of 2 kg/cm^2^. After locating the pain points, the doctor will use a black pen to draw “×” on it.

#### The stuck-moving needle acupuncture method

The patient will take a seated position during acupuncture. After routine disinfection on the selected myofascial trigger points (MTrp), the skin will be cleaned with alcohol (75%). The applicator will tighten the patient’s skin with his or her left hand and will hold the stuck-moving needle (specification, 0.40 × 40 mm) in his or her right hand (the position of the needle is approximately 1 cm from the needle tip), and the stuck-moving needle will be inserted perpendicularly into the Trp (the degree of convulsions after the patient’s pain is triggered). Further, a needle will be twisted clockwise slowly (90–180°) until the muscle fibers are wounded and until the needle becomes tight and twisted. At this point, patient may describe pain and astringency. Then, the needle will be lifted again with 3 to 5 times by shaking. The strength of lifting the needle will depend on the patient’s condition and acupuncture position. Furthermore, a needle should be ejected quickly. While lifting the needle, the doctor will twist needle counter clockwise until the muscle fibers are relaxed. After the treatment, the patient will be supplemented with physical exercises. The exercise method is same as provided to group C.

##### Treatment course

One treatment every other day, three treatments per week, total of 9 treatments for 3 weeks.

### Group B: common acupuncture control group

#### Acupoints

Jianyu (LI15), Jianliao (TE 14), Jianzhen (SI 9), Biru (LI14), Ashi points.

#### Method of operation

The patient will be taken the lateral position and exposes the affected side of the shoulder. After routine sterilization of the acupuncture site, a milli needle with a diameter of 0.25 mm and a length of 40–50 mm will be used. The acupoint locations are based on the “2006 People’s Republic of China National Standard” (GB/T1234–2006), and the acupuncture operation is based on the textbook of the 12th Five-Year Plan of the Ministry of Health. The skin will be cleaned with alcohol (75%). Then, needle will be quickly pierced into acupoints with the depth of 1.9–2.6 in., and twirling the needle until “De qi”. After using the mild reinforcing-attenuating method, the needle is retained for 30 min. After daily acupuncture, exercise will be provided which is as same as group C.

#### Treatment

One treatment every other day, 3 times a week, 9 times for 3 weeks.

### Group C: physical exercise control group

The physical exercise method of the shoulder joint is carried out according to the physical exercise program recommended in the Evidence-based clinical guidelines for the diagnosis, “The Assessment and Physiotherapy Management of Contracted (frozen) Shoulder” prepared by Hanchard et al. [13] in 2012. The patient’s training will be consisted of 12 sports units, including active and passive training, for a total of 50 min. Each workout will have a professional physiotherapist (Cheng Maomao physical therapist) to guide the treatment and provide a practical exercise program for the patient. Treatment will take place 3 times a week for 3 weeks for a total of 9 treatments. The basic movements are as follows: active shoulder joint exercise with bare hands, let the patient use the diseased shoulder joint as the axis of shoulder joint movement, and do small rotation movements of the shoulder joint. First, rotate clockwise for 4 min, then counterclockwise for 4 min. Subsequently, the intensity can be increased; the two movements alternated for a total of 20 min. In the later stage, strength of exercise will be increased with the help of the physiotherapist, and the passive movement will be carried out for 10 min. Then, it will be used for shoulder flexion, abduction, extension and internal rotation, external rotation, and other actions by means of assistive devices such as shoulder rotation training device and fitness ball technique. Finally, the standing training exercises will be carried out for the spine and side bends of the spine.

### Measures to improve patient adherence

On a voluntary basis with the signed informed consent, patient’s adherence will be improved using the subject manual and Short Messaging Service (SMS). The acupuncturists will possess at least 5 years of clinical experience in acupuncture practice. This is to ensure that the treatments will be administered consistently.

## Outcomes

The following outcomes will be assessed by independent outcome assessors. These assessors will be trained before participating in this trial and unaware of the group assignments. The outcome assessments will be performed at four time points including at the baseline, at the end of the stuck-moving needle acupuncture treatments, in the 4-week follow-up period, and at the end of the follow-up period.

### Main outcome indicators

The primary evaluation index in the trial will be the change involved in the scores on the Visual Analogue Scale (VAS) and measurement of range of joint motion (ROM) from the baseline period through the 12 weeks of the follow-up period.

#### Visual Analogue Scale (VAS)

The VAS for pain assessment is widely used in China. The basic method is to use a 10-cm-long swimming ruler which has ten-scale on one side and the two ends of the ruler are with the digits 0 and 10. “0 point” indicates no pain, and “10 point” represents the most severe pain which is unbearable. During the assessment, the scaled side is faced back to the patient, and the patient marks on the ruler with the scale points that represents his or her pain level, and the physician records the score based on the position marked by the patient.

#### Measurement of range of joint motion (ROM)

The degree of mobility of the shoulder joint was measured using the American muscle strength test and the joint mobility meter (portable) (Hoggan MicroFET3). This study will measure the angle of the shoulder flexion, extension, abduction, internal rotation, and external rotation.

### Secondary outcome

#### Measurement of pressure pain threshold (PPT) and pressure pain tolerance (PTT)

This study will be performed using the FDK20 handheld pressure an algeometer manufactured by Wagner Instruments, USA. The device measures the individual’s pain sensitivity by applying pressure which includes measuring the pressure pain threshold (PPT) and the pressure pain tolerance (PTT). Initially, the data that will be transferred to the tester when the subject just felt pain, which will be recorded as PPT. Furthermore, after a short pause, the pressure will be increased until the pain becomes intolerable to the subject. After a short pause, the tester may increase the pressure until the patient feels that the pain caused by this pressure is intolerable. When the patient informs the tester for the second time, the tester will record it as PTT. The test value of the pain indicator is only visible to the tester during the whole process. The tester sequentially measures the pain threshold and pain tolerance threshold of the patient’s marked points, and the test interval for different points is 5 min. The data will be analyzed in units of kg/cm^2^.

#### Oxford Shoulder Score (OSS)

The OSS consisted of 12 questions. These questions were related to the pain (1~4 questions) and functional activities (5~12 questions). There are five alternative answers for each question. The best case is 1 point, the worst is 5 points, and the total score will be between 12 and 60 points. The higher the score, the worse the shoulder function will be.

#### Short form-36 questionnaire (SF-36)

The quality of life assessment scale includes 36 questions, which are related to the general health problems, physical function problems, limitations of daily life due to physical strength and physical pain, social skills, psychological repression and well-being, and functional limitations caused by emotional problems.

#### Evaluation of patient’s satisfaction

Based on the present study, this scale will provide medical services to patients, which will allow evaluation of treatment in a true and objective manner.

### Sample size estimation

According to the previous researches, the difference between the VAS score before and after treatment in the general acupuncture group was 4.7, with a standard deviation of 1, whereas the difference in VAS score before and after treatment in the functional exercise group was 3.5, with a standard deviation of 1 [14]. In combination with the pilot experimental results from this study, the VAS score before and after treatment of frozen shoulder patients with stuck-moving needle acupuncture dropped by 5.2 and the standard deviation was 1.06. This study was a prospective study with three groups and the sample size as 1:1:1. The sample size was calculated using *F*-test in software SAS 9.4, and we used single-factor analysis of variance to calculate the sample size. The test level was set at *α* = 0.05, β = 0.1, and the test efficiency was set at 1 − *β* = 0.9. The results showed that the test power was 0.931, and the number of samples in each group was not less than 30. By considering the problem of missing samples or losing to follow-up, the sample size will be increased by 20. This means that the minimum sample size of each group will be 36 patients, and finally, the total sample of the three groups will include cases not less than 108.

### Adverse events

All adverse events that will occur during the study will be recorded. The records will include the description of these events, start time, severity and frequency of occurrence, end time, ongoing treatment, results, and the track and analysis.

1. A serious adverse event (SAE) is an adverse event in which any of the following circumstances can occur.
  1. An event that causes the patient to die or endanger the patient’s life.
  2. The events that result in permanent or significant disability or loss of function in the patient.
  3. The events that lead to hospitalization.
  4. The important medical events need to use drugs or surgery to prevent serious consequences.
2. Treatment of serious adverse events

Once a serious adverse event is found, the study physician must report to the study leader and the study team. The study team should report to the Ethics Committee and the responsible units within 24 h and record the serious adverse events in the original data. Original data may include carefully filling out the adverse event report form, recording the start time, the severity, the final outcome, the relationship with acupuncture treatment, the details of the treatment, and should immediately consult the relevant departments to take the necessary treatment.

The trial of a SAE patient will be discontinued and become a withdraw subject, while all costs for handling adverse events will be covered by this study.

### Safety evaluation

The project will evaluate the safety of vital signs and the incidence of adverse acupuncture reactions.

1. Vital signs: Post-treatment respiration, heart rate, blood pressure, and body temperature.
2. Incidence of adverse events: Adverse events will be referred as any unintended symptoms, signs, or health conditions that occurred after the start of the study. However, adverse events will not have a relationship with the treatment of this study. Adverse events include dizziness, hematoma, bleeding, infection, and abnormal pain at the treatment site. The number of adverse reactions will be counted at the end of the study, divided by the total number of each group, and the analysis will be carried out for the adverse events.
3. Compliance analysis: The patient’s compliance with the treatment will be counted by the number of treatments. The formula is as follows: treatment compliance = the number of treatments the patient has received/the number of treatments the patient should receive × 100%.

### Statistical analysis

All statistical analysis will be carried out by the professionals using SPSS (v21.0) software. The participants in the statistical analysis will be unaware as well for the specific grouping content of the trial. This study will use a full analysis set (FAS) and per protocol set (PPS), and the final conclusion will be based on FAS. The full analysis set (FAS) is based on the intent to treatment (ITT). Patients who participate in randomization and receive at least one treatment will be included in the analysis. In the FAS analysis, for patients who do not complete the trial, the main indicators of the missing will be derived from the Last Observation Carried Forward (LOCF). Continuous variables will be presented as mean ± standard deviation (normally distributed data) or medians and ranges (non-normally distributed data). Frequencies and percentages will be used to count the data. All statistics will be performed by bilateral differential test, with *P* value < 0.05 considered statistically significant.

For the balance of basic values, analysis of variance or chi-square test or rank sum test will be used. For the primary and secondary outcome, the analysis of variance will be used. If confounding factors have an impact on the results, then the co-variance analysis will be used. For the safety analysis, the incidence of three groups of adverse events will be compared using a chi-square test, and the adverse events that will occur in this trial will be listed. At the same time, the paired *t* test will be used to compare the differences between the groups before and after treatment. The comparison among the three groups before and after treatment will be analyzed by variance. The rate changes before and after treatment in the three groups will be measured by chi-square test or non-parametric test.

### Quality control

1. This study will use a randomized control design, and the randomization protocol will be held by the management team of the study team who do not participate in the project to ensure random allocation and control biases.
2. This study is limited to the inclusion criteria and will establish a clear inclusion and exclusion criteria to control biases.
3. This study will use blind method evaluation and will evaluate the efficacy and safety by evaluators who do not know the grouping situation to ensure the reliability of the research results.
4. During the study, the pain tester, muscle strength, and joint mobility test will be carried out according to various standard operating procedures to control biases.
5. The quality control inspection of this study will be conducted by the researcher to designate at least one non-quality implementer to conduct a quality check on the research of each center of the subject to control the bias.

### Quality assurance

Initial meeting of the project will be held to conduct unified training. Key training will be carried out on the project implementation plan and SOP, so that each participant will master the research process and specific implementation rules to ensure the reliability of the conclusion. During the study, each center will be supervised regularly to control biases.

### Data collection and management

Paper CRFs will be used to record all the information required from the protocol which will be collected from the hospital. There will be an instructions page at the start of each postal questionnaire and for the more complex hospital CRFs. Each trial participant will have a unique identification number that will be pre-recorded on all CRFs. Initially, the data will be listed on the CRF form, and after interviewing 20 participants, the data of clinical research will be input in a group of 20 cases. Besides, the input approach will be double-input, which will be input by two data input personnel without contact. Meanwhile, the data manager will compare the differences with the initial data and fill in the reasons for it. During the trial, a monthly data inspection will be needed, and the content involves data assembled by every center abnormal data, and delayed data in progress tracking. The data input in the paper type of CRF form will be received from the primary data. Furthermore, the researcher will preserve the copies of the test-related data, the data of the subject, and all other initial data until the announcement from the team leader to discard them. Moreover, researchers at the center should obey the rule of confidentiality of the trial. After the primary data which will be held for 3 years, non-research team members cannot browse and borrow it.

### Data monitoring and auditing

As this test is a multi-center study, therefore, before the start of each research center, the team leader from every unit will monitor the research center. They will check whether the research-related facilities and equipment are complete and well prepared, and whether or not the researchers are proficient in the test operation process and equipment use. The chief investigator of the team leader unit will inform each clinical center investigator of their responsibilities, and how to correctly and completely fill in the relevant documents for the trial. Through the investigator training session, the investigator and his team will understand the trial plan, completing the CRF form, use of the evaluation tools, as well as the test procedures. Then the chief investigator of the team leader unit will visit the start-up status of each center. During the test, the third-party data management staff will regularly and strictly monitor the filling of the CRF form. The third-party data management staff will mainly monitor whether the researcher screens the subjects in accordance with the admission criteria. During the inspection, the CRF form will be checked if it is accurate, directly with reference to the original data.

### Informed consent

1. Before conducting a conversation with the subject, the researcher should understand and check whether the informed consent is comprehensive, whether it meets the ethical requirements, and whether it meets the rights and obligations of the subjects as specified in the “Helsinki Declaration.”
2. Explain the entire contents of the informed consent form to the subject in a simple and understandable language. The subject will voluntarily participate in the clinical trial and has the right to withdraw at any stage of the trial without discrimination and retaliation. The personal data and information of the participants participating in the trial will be kept confidential, and subjects may be assigned to different trial groups to take different treatments. Informed consent should be signed by the subject or other legal representative.

### Patient dropouts

Investigators will have the authority to terminate patient participation at any time if the investigator deems it is in the best interests of the patient. Termination criteria are as follows: (1) Patients with serious adverse reactions or combined with other critical illnesses in the study. (2) The subject asks to withdraw from the trial during the course of treatment and persuasion is ineffective. (3) Subjects who are not cooperating to the treatment and persuasion are ineffective by the doctor. (4) If the subject receives half of the treatment, then he or she should be included in the statistical analysis of efficacy.

### Post-trial care

This study will not provide any post-trial care.

### Protocol amendments

If the protocol changes during the implementation of the study, researchers will communicate the important protocol modifications (e.g., changes to eligibility criteria, outcomes, analyses) to the relevant parties, including the fund regulator, trial participants, trial registries, and journals.

### Confidentiality

This study follows the ethical principles set out in the “Helsinki Declaration” and the various laws and regulations related to clinical research in China. Patients may receive acupuncture treatments such as trigger points during the study. Moreover, patients will receive free color Doppler ultrasound, muscle strength measurement, pain threshold measurement, infrared thermography, and various evaluation tests. Some patients may experience fainting, pain, bleeding, hematoma, or infection during acupuncture treatment. When such circumstance will arise, the patient will be handled properly and recorded in a timely manner.

## Discussion

Idiopathic frozen shoulder is a common and painful disease with a long course. Although the disease has a certain tendency of self-healing, it lasts for prolonged time. It is difficult for the affected side of shoulder joint to fully return in normal function. If it does not function properly, it may lead to functional disability and directly affects the day-today life of the patient. Worldwide, chronic shoulder pain is considered as one of the indications as most amenable to treat with acupuncture [26, 27]. According to the results of previous systematic reviews and clinical studies [28, 29], acupuncture as a main treatment for frozen shoulders has a good therapeutic effect, and the key factors affecting the effect of acupuncture are acupuncture techniques and acupuncture sites. The acupuncture method used in this study is stuck-moving needle acupuncture therapy. The acupuncture sites are selected from the local MTrp of the shoulder joint. At present, there is no significant clinical research which can prove whether the combination of the stuck-moving needle and MTrp is better than the combination of ordinary acupuncture and the acupuncture point. Our pilot study showed that stuck-moving needle used for acupuncture MTrp was effective in improving the pain and movement limitation of the patients with idiopathic frozen shoulder. However, this study was a single case observation in a single center, without a control group. Therefore, in our study, we designed a multi-center, large-sample, randomized controlled clinical trial to evaluate the efficacy and safety of stuck-moving needle acupuncture the MTrp in improving pain and range of motion in patients with idiopathic frozen shoulder. The ultimate aim of the study was to select an effective acupuncture therapy for patients with idiopathic frozen shoulder.

Stuck-moving needle therapy is derived from traditional acupuncture therapy, clinical practices, learning from needles (e.g., crochet needles, loose needles, floating needles, minimally invasive needles, and many other needles), massage, and acupoint techniques with multiple treatment effects in one. In addition, it has multi-angle and multi-directional treatment effects, especially for stiffness, swelling, adhesion contracture, and nodular tissue decompression and reduction. It is considered to be the perfect combination of traditional acupuncture theory and modern medical theory, as well as minimally invasive technology. Most important point which distinguishes stuck-moving needle therapy from other therapies and generating unique curative effects is its dynamic nature. Initially, the needle applicator applies active motion to the needles fixed at the treatment site to achieve a benign, suitable, and effective needle volume to produce “dynamic needle wave,” which is the key to the needle effect. Then, the operator is assisted by auxiliary movements. During the process of acupuncture in the right hand, the left hand moderately lifts the operation site. The third one is the cooperative movement of the patient [20]. In the process of applying the needle, the doctor may guide the patient to perform appropriate activities. In recent years, stuck-moving needle therapy is widely used in clinical practices and exhibited promising effects in treating patients with joint and muscle pain [30–33].

The needle application site is a key factor that affects the effect of acupuncture. In this study, the operation site of the stuck-moving needle is the localized MTrp of the shoulder joint. Studies in recent years have found that the occurrence of frozen shoulders is often related to the muscles around the shoulder joint related to the pain points [34, 35]. These muscles include deltoid muscle, biceps, pectoralis major, pectoralis minor, and coracobrachialis muscle. These points of muscle are common, and it can cause pain and impair shoulder movement. If it can be accurately positioned in clinical treatment for effective treatment, the effect will be greatly improved. The pain points of the affected muscles of the shoulder joint are characterized by tenderness, which restricts the complete extension of the muscles, causing muscle weakness, and can be directly compressed to conduct the patient’s identifiable pain, whereas sufficient stimulation can cause twitch response. Based on the characteristics of MTrp with tenderness and referred pain [36], we used a pain meter to determine the location of the local pain points in the shoulder joint and then performed a stuck-moving needle operation with a marker to ensure the accuracy of the operation. During this study, we used patented stuck-moving needles termed as Li Zhenquan’s needles, produced by Suzhou Acupuncture Supplies Co., Ltd., with a size of 0.4 × 40 mm.

The stuck-moving needle acupuncture method can increase the stimulation intensity, breadth, and depth of acupuncture. It is applied to the Trp for the localized activation of the shoulder joint, making the local capillaries of the Trp quickly contract and relax, improve the state of local tissue microcirculation, and accelerate the local metabolism. At the same time, by increasing the intensity of acupuncture, it can increase the rapid release of certain analgesics in the pain point. This relieves pain symptoms.

The setting of control group also plays an important role in the efficacy evaluation. It is the basis of accurate evaluation of curative effect. According to the WHO published guidelines for clinical research in acupuncture and moxibustion requirements for the establishment of control groups in clinical studies of acupuncture and moxibustion, we set up two control groups. Exercise programs are the basic treatment and also a control group. The common acupuncture group is a positive control group, and the stuck-moving needle was used to acupuncture MTrp as the treatment group, to verify the effectiveness of treatment group.

The selection of appropriate outcome is crucial when designing clinical trials. A review of shoulder pain trials found that trialists reported pain in 87%, function in 72%, range of motion in 67%, adverse events in 27%, patient-reported treatment success in 24%, strength in 18%, and health-related quality of life in 18% [37]. Therefore, we choose primary outcomes including a measure of pain (VAS) and range of motion (ROM), whereas secondary outcome included a measure of pain (PTT and PPT), function (OSS), health-related quality of life (SF-36), patient satisfaction evaluation, and adverse events. The measurement time was from baseline through the 12-week follow-up period.

However, this study also has its limitation. First, the implementation of the blind method is difficult due to the nature of acupuncture, and the masking of acupuncturists is quite difficult to achieve. However, the statistician will be masked. Second, the follow-up time is too short, probably due to the current status of research funding and research time, and patients were followed up for a period of 12 weeks after the end of treatment in this study. Follow-up was performed at week 4 and week 12. Frozen shoulder is a chronic degenerative osteoarthropathy with a long course and the high possibility of recurrence. Therefore, for the future trial design, the follow-up time should be appropriately extended and comprehensive evaluation should be carried out with the long-term treatment effect for frozen shoulder by the stagnant acupuncture method. Nonetheless, the trials carried out in this study and subsequent results will provide more reliable evidence for clinical treatment of FS.

## Trial status

Protocol version V1.1 dated 2 Dec. 2019. The first participant was enrolled on 02 Jan. 2020. Recruitment will be complete on 30 Jan. 2021.

### Supplementary information

The study protocol has been reported in accordance with the Standard Protocol: Recommendations for Interventional Trials (SPIRIT) guidelines (Additional file 1).

## Supplementary information

**Additional file 1.** SPIRIT 2013 Checklist: recommended items to address in a clinical trial protocol and related documents.

## Data Availability

The datasets generated and/or analyzed are available from the corresponding author upon reasonable request.

## Abbreviations

FS: Frozen shoulder
CRF: Case report form
VAS: Visual Analogue Scale
ROM: Measurement of range of joint motion
PPT: Pressure pain threshold
PTT: Pressure pain tolerance
OSS: Oxford Shoulder Score
SF-36: 36-item short form survey
TCM: Traditional Chinese medicine
SMS: Short Messaging Service
